# Serum cystatin C is an early renal dysfunction biomarker in patients with hepatitis C virus

**DOI:** 10.1186/s43066-022-00231-x

**Published:** 2022-11-24

**Authors:** Nagwa Mohamed Assem, Amany Ibrahim Mohammed, Hamed Mohamed Abdel Barry, Ibrahim El Tantawy El Sayed, Ibrahim Elmadbouh

**Affiliations:** 1 Department of Biochemistry, Medical Research Institute, Alexandria University, Alexandria, Egypt; 2 Chemistry Department, Faculty of Science, Menoufia University, Shebin El Kom, Egypt; 3 Department of Medical Biochemistry and Molecular Biology, Faculty of Medicine, Menoufia University, Shebin El Kom, Egypt

**Keywords:** HCV, AKI, Cryoglobulins, Cystatin C, Complement C3, RF, PCR

## Abstract

**Background:**

Hepatitis C virus (HCV) may induce extrahepatic manifestations as acute or chronic renal dysfunction. The aim was to evaluate the diagnostic role of some biomarkers as cystatin C, cryoglobulins, rheumatoid factor (RF), and complement C3 for extrahepatic renal affection in newly diagnosed patients with HCV infection.

**Methods:**

Blood and urine were collected from randomized individuals screened for new HCV infection (*n*=400). The studied populations were divided into 3 groups: control group I: thirty healthy individuals not suffering from either liver or kidney diseases, group IIa: thirty HCV patients who have positive HCV antibody test but showed negative PCR test, and group IIb: thirty HCV patients who showed positive results for both HCV antibody and PCR tests.

**Results:**

In HCV group IIb, levels of serum total bilirubin, AST and ALT, and urine albumin/creatinine ratio were increased whereas serum albumin and creatinine clearance were decreased versus other groups. However, the levels of blood urea nitrogen and serum creatinine were still within the normal range in all groups. In HCV group IIb, cystatin C, cryoglobulins, and RF levels were increased; meanwhile, serum creatinine/cystatin C ratio and complement 3 levels were decreased compared to the other groups. HCV-infected patients significantly had higher serum cystatin C (>1.24 mg/L, *P*<0.001) and lower creatinine/cystatin C ratio (<70.1μMol/mg, *P*=0.002), and cystatin C was significantly correlated with liver and kidney parameters.

**Conclusion:**

High serum cystatin C and low creatinine/cystatin C ratio may be early indicators of mild renal dysfunction with normal serum levels of creatinine in HCV-infected individuals.

## Introduction

Acute hepatitis C virus (HCV) is a widespread infectious disease that affects the liver in about 200 million people worldwide [1] and progresses to chronic HCV in 50–80% of patients that may be leading to liver fibrosis, cirrhosis, hepatocellular carcinoma, and death [2]. However, there are also extrahepatic manifestations of chronic HCV which include glomerulonephritis, thyroiditis, insulin resistance, diabetes mellitus, porphyria cutanea tarda, lichen planus, vitiligo, seronegative arthritis, cryoglobulinemia, and lymphoproliferative disorders [3].

HCV-infected patients may present with acute kidney injury (AKI), chronic kidney disease (CKD), and end-stage renal disease (ESRD) within 5 years [3, 4]. Blood urea nitrogen (BUN), creatinine, and creatinine clearance (as an estimation of glomerular filtration rate, eGFR) are not sensitive or accurate for early kidney dysfunction (AKI) because these markers depend on diet and body metabolic and muscle condition [5–7].

Cystatin C is a potent inhibitor of lysosomal proteinases and probably one of the most important extracellular inhibitors of cysteine proteases [8–10]. Cystatin C has an advantage as low molecular weight (13.3 kilodaltons), produced at a constant rate in all nucleated cells, eliminated by glomerular filtration, reabsorbed, and catalyzed in renal proximal tubular cells [11]. Serum levels of cystatin C are independent of age, sex, and muscle mass and are not influenced by bilirubinemia, inflammation, or neoplasia [12]. Therefore, serum cystatin C could provide an alternative method to creatinine-based criteria for eGFR [12, 13]. Moreover, serum cystatin C has some advantages over the serum creatinine as a biomarker for eGFR [5], and it is elevated in early hepatic fibrosis [8] rather than early kidney disease [12].

Interactions between HCV and the host immune system may play an important role in the viral persistence of chronic HCV patients. The presence of extrahepatic parameters such as complement C3 [14, 15] and rheumatoid factor [16], cryoglobulins, and mixed cryoglobulins and cystatin C may be involved in renal injury [17].

The aim was to evaluate the diagnostic role of cystatin C and creatinine/cystatin C ratio, cryoglobulins, rheumatoid factor (RF), and complement C3 as early biomarkers of extrahepatic kidney dysfunction in newly diagnosed patients with HCV.

## Material and methods

### Patients

All subjects were selected from Menoufia University Hospital within the national campaign to combat virus C. Blood samples were taken from the brachial vein from all subjects and their laboratory analysis was done in Menoufia University Hospital and Medical Biochemistry Department, Faculty of Medicine, Menoufia University. Hospital’s Review Board has given ethics approval and all participants have written an informed consent prior to subject characterization and sample collections in accordance with the guidelines of the Declaration of Helsinki.

For the inclusion criteria, this cross-section study was performed on individuals (*n* = 400) in a health screening program for HCV infection between 2019 and 2021; all were males with age 35–45 years according to the results of hepatitis C virus antibody and PCR; the studied population was divided into 3 groups as follows: group I: 190 individuals who showed a negative result for HCV antibody test and 30 healthy individuals not suffering from both liver and kidney disease according to liver and kidney function parameter tests were chosen as a control group. HCV group II: 210 individuals showed a positive result for the HCV antibody test and were classified according to the results of the polymerase chain reaction (PCR) test for them. The result of PCR was as follows: 95 individuals showed a negative result for the PCR test and 115 individuals showed a positive result for the PCR test; 30 individuals from each of subgroup II not suffering from apparent kidney disease were chosen to be involved in this study, as group IIa and group IIb, respectively.

The exclusion criteria were hypertension, diabetes millets, pre-existing kidney disease, established liver cirrhosis of different or mixed etiologies such as alcohol intake, hepatitis B, autoimmune liver disease, non-alcoholic fatty liver disease (NAFLD), hepatocellular carcinoma, abnormal thyroid function, or malignant diseases.

### Laboratory analysis

The blood samples (10 mL) were centrifuged (4000 rpm for 10 min) for the collection of serum. Each serum sample was divided into two parts. The first part was directly stored at −80°C until assayed for quantitative estimation of HCV antibody levels and PCR assay. In the second part, serum samples were stored at −20°C for biochemical investigations. All serums were thawed at room temperature when ready for analysis. Urine 24-h samples were collected in a sterile plastic container for measuring urine creatinine and albumin

### Determination of liver and renal function tests

Serum total bilirubin, aspartate transaminase (AST), alanine transaminase (ALT), albumin, creatinine, and blood urea nitrogen (BUN) were determined using commercial kits on the Cobas e501 analyzer (Roche Diagnostics, Germany).

Urine albumin and creatinine were measured using commercial kits on the Cobas e501 analyzer (Roche Diagnostics, Germany) for urine albumin to creatine ratio (30 mg/g or greater detection of albuminuria) [18].

The creatinine clearance was calculated from the formula: urinary creatinine concentration (U) (mg/mL) × urine volume (V) (mL/min)/serum creatinine concentration (P) (mg/mL) [18]. The estimated creatinine clearance is not normally physiologically greater than 120 mL/min for most adults.

### Determination of HCV antibody concentration

Anti-HCV antibodies were determined using electrochemiluminescence immunoassay “ELISA” using Cobas 6000 (ROCHE, Germany). The assay procedure was carried out in a microwell coated with a combination of recombinant hepatitis C virus (rHCV) antigen (c22-3, c200, and NS5).

### Determination of RT-qPCR for HCV RNA

The procedure involved 3 main steps: HCV RNA extraction, conversion of HCV RNA to complementary DNA (cDNA), and amplification and detection of the amplified products. HCV RNA extraction was performed by using an Artus® HCV RG RT-PCR kit (Qiagen GmbH, Germany, Cat No. 4518263) according to the manufacturer’s instructions. HCV levels were determined using the Rotor-Gene Q MDx (Rotor-Gene Q MDx, Qiagen, Germany) Light Cycler Real-Time PCR System using Rotor-Gene-3000 software version 6.0.23 under the following conditions: 50°C, 25 min; 94°C, 2 min; 5 cycles of 94°C for 10 s, 55°C for 15 s, and 72°C for 15 s; and 42 cycles of 94°C for 10 s, 60°C for 45 s, and 40°C for 30 s. Fluorescence was measured at 60°C for each cycle. An internal quality control serum was included during RT-PCR.

### Determination of serum cryoglobulins

Cryoglobulins were determined by human enzyme-linked immunosorbent assay (ELISA) according to the manufacturer’s instructions (MyBioSource, San Diego, CA, USA).

### Determination of serum complement C3

Serum complement component 3 (C3) levels were measured by the human ELISA technique (ab108823) (Abcam, Cambridge, MA, USA). Normal ranges of serum C3 levels were set at 80–160 mg/dL [19].

### Determination of serum rheumatoid factor (RF)

Serum rheumatoid factor IgM were assayed by human rheumatoid factor IgM ELISA (Cell BioLabs Inc. USA) using reagents and controls supplied by the manufacturer with results considered positive when they exceeded values of 12 IU/mL [20].

### Determination of serum cystatin C

Serum cystatin C was measured using a human ELISA assay (BioVender, Brno, Czech Republic). The creatinine/cystatin C ratio was calculated as the serum creatinine concentration (μMol/L) divided by the cystatin C concentration (mg/L) [21, 22].

### Statistical analysis

The data were analyzed by SPSS statistical package version 20 on an IBM-compatible computer. Quantitative data were expressed as mean ± standard deviation and analyzed by applying Student’s *t-*test, one-way ANOVA, and Tukey post hoc tests to determine significant differences among all groups. ROC curve was used to determine cutoff points, sensitivity %, and specificity % for quantitative variables of interest. Pearson correlation (*r*) was used to assess the correlation between variables’ parameters. A *P* value of <0.05 was considered as being statistically significant.

## Results

The laboratory results of HCV-infected patients (groups IIa and IIb) indicated a significantly higher serum total bilirubin, ALT, AST, BUN, creatinine, and urine albumin/creatinine ratio and a significantly lower serum albumin and creatinine clearance concentration as compared to the controls (Table 1). Despite serum creatinine and BUN levels were increased in both subgroups (IIa, IIb), as compared to the controls, these results are still within the normal range (Table 1).

**Table 1:** Laboratory data between the three studied groups

	Control (group I) ***n*** = 30	HCV-positive antibodies (group II)		Test ***P*** value	P1 P2 P3
		Group IIa (negative PCR) ***n*** = 30	Group IIb (positive PCR) ***n***= 30		
**Liver and renal function test**					
**Serum total bilirubin (mg/dL)**	0.59 ± 0.13	0.79 ± 0.12	1.10 ± 0.22	ANOVA=21.3 0.0001*	0.001* 0.002* 0.005*
**Serum alanine transaminase (ALT) (U/L)**	24.67 ± 6.55	40.17 ± 9.62	50.77 ± 9.74	ANOVA=28.62 0.001*	0.0025* 0.0014* 0.004*
**Serum aspartate transaminase (AST) (U/L)**	25.13 ± 4.50	33.93 ± 6.65	46.13 ± 9.37	ANOVA=17.85 0.001*	0.015* 0.0025* 0.0036*
**Serum albumin (g/dL)**	4.74 ± 0.41	3.73 ± 0.32	3.36 ± 0.52	ANOVA=25.6 0.0001*	0.0016* 0.008* 0.001*
**Blood urea nitrogen (BUN) (mg/dL)**	10.53 ± 1.96	10.53 ± 1.41	11.93 ± 2.03	ANOVA=19.85 0.001*	0.500 N.S. 0.004* 0.0014*
**Serum creatinine (mg/dL or μMol/L)**	0.76 ± 0.10 67.18 ± 8.51	0.86 ± 0.13 76.15 ± 11.32	0.98 ± 0.22 86.41 ± 19.59	ANOVA=24.2 0.0002*	0.0049* 0.006* 0.0179*
**Creatinine clearance (mL/min)**	116.67 ± 12.72	103.33 ± 16.83	89.20 ± 12.90	ANOVA=14.52 0.006*	0.085 N.S. 0.037* 0.00028*
**Urine albumin/creatinine ratio (mg/g)**	19.30 ± 3.04	27.04 ± 4.71	30.40 ± 6.17	ANOVA=36.52 0.0001*	0.0011* 0.0031* 0.0104*
**Extrahepatic renal affection parameters**					
**Serum cryoglobulins (ng/mL)**	N.D.	125.07 ± 25.7	240.63 ± 11.27	*T* = 6.81 0.0001*	
**Serum complement C3 (mg/dL)**	133.43 ± 24.42	89.57 ± 9.88	68.37 ± 7.77	ANOVA=33.6 0.0001*	0.001* 0.001* 0.011*
**Serum rheumatoid factor (IU/mL)**	7.75 ± 2.02	31.28 ± 19.01	94.92 ± 31.22	ANOVA=42.1 0.0001*	0.0001* 0.0001* 0.004*
**Serum cystatin C (mg/L)**	0.66 ± 0.22	1.19 ± 0.22	1.57 ± 0.31	ANOVA=19.25 0.001*	0.002* 0.001* 0.006*
**Serum creatinine/cystatin C ratio (μMol/mg)**	99.60 ± 31.03	70.98 ± 16.67	55.07 ± 17.25	ANOVA=27.9 <0.001*	<0.001* <0.001* 0.03*

P1: comparison between the control group and group IIa. P2: comparison between the control group and group IIb. P3: comparison between groups IIa and IIb

^*^P was significant if < 0.05

The present study showed that HCV infection caused alterations in some extrahepatic parameters such as cryoglobulin formation in both groups IIa and group IIb where cryoglobulins were not detected in the controls (Table 1). Also, in both group IIa and group IIb, our data indicated a significantly increased rheumatoid factor and cystatin C and a significant decrease in complement C3 and creatinine/cystatin C ratio versus controls (Table 1).

**Fig. 1:**
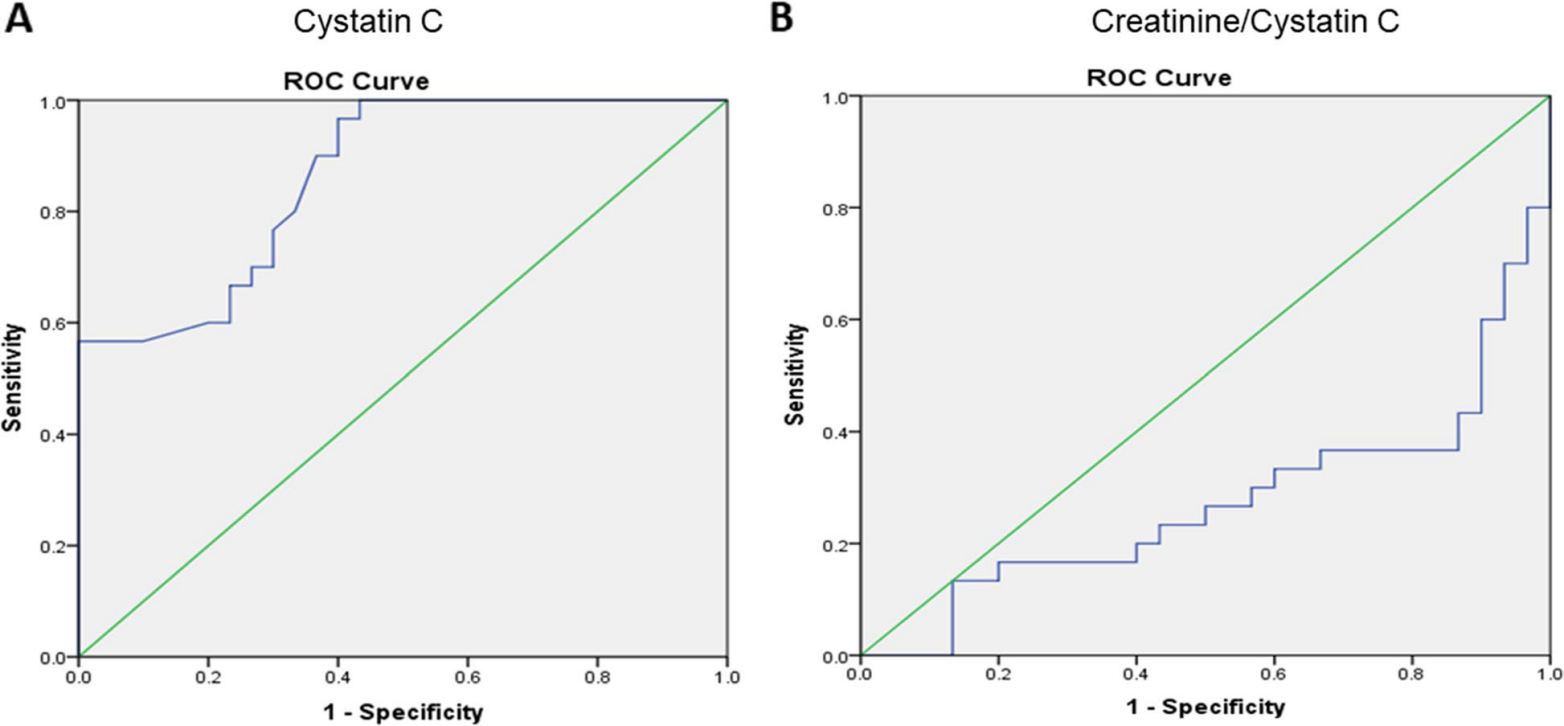
ROC curves of cystatin C in HCV-infected patients (**A**). ROC curves of creatinine/cystatin C in HCV-infected patients (**B**)

HCV-infected patients had significantly higher serum cystatin C with cutoff value (> 1.24 mg/L, *P* <0.001) and lower creatinine/cystatin C ratio (< 70.1 μMol/mg, *P* = 0.002). Also, the area under the curve (AUC), 95% CI, sensitivity %, and specificity % are assessed and shown in Table 2 and Fig. 1.

**Table 2:** ROC curve, sensitivity, specificity, and accuracy of cystatin C and creatinine/cystatin C ratio levels to determine susceptibility and risk of kidney injury among groups

	Cystatin C (mg/L)	Creatinine/cystatin C ratio (μMol/mg)
**AUC**	0.864	0.272
***P*value**	<0.001*	0.002*
**95% CI**	0.775–0.952	0.141–0.404
**Cutoff point**	1.24	70.1
**Sensitivity**	76.7 %	23.3%
**Specificity**	70%	56.7%

*AUC* area under the curve, *CI* confidence interval

Serum cystatin C was positively correlated with serum total bilirubin, ALT, AST, BUN, creatinine, cryoglobulins, RF, and urine albumin/creatinine ratio and was negatively correlated with serum albumin and creatinine clearance, complement C3 and creatinine/cystatin C ratio as shown in Table 3. Also, serum creatinine/cystatin C ratio was negatively correlated with serum total bilirubin, ALT, AST, cryoglobulins, RF, and urine albumin/creatinine ratio and was positively correlated with serum albumin and complement C3 as shown in Table 3.

**Table 3:** Correlation between variables

		Cystatin C (mg/L)	Creatinine/cystatin C (μMol/mg)
**Serum total bilirubin (mg/dL)**	*r*=	0.711	− 0.418
	*P* value	0.001*	0.001*
**Serum alanine transaminase (ALT) (U/L)**	*r*=	0.720	− 0.516
	*P* value	0.001*	0.001*
**Serum aspartate transaminase (AST) (U/L)**	*r*=	0.741	− 0.559
	*P* value	0.001*	0.001*
**Serum albumin (g/dL)**	*r*=	−0.647	0.444
	*P* value	0.001*	0.001*
**Blood urea nitrogen (BUN) (mg/dL)**	*r*=	0.249	− 0.077
	*P* value	0.018*	.469
**Creatinine (mg/dL)**	*r*=	0.454	0.029
	*P* value	0.001*	0.786
**Creatinine clearance (mL/min)**	*r*=	−0.186	0.159
	*P* value	0.079	0.134
**Urine albumin/creatinine ratio (mg/g)**	*r*=	0.678	− 0.488
	*P* value	0.001*	0.001*
**Serum cryoglobulins (ng/mL)**	*r*=	0.446	− 0.374
	*P* value	0.011*	0.003*
**Complement C3 (mg/dL)**	*r*=	− 0.435	0.527
	*P* value	0.018*	0.001*
**Rheumatoid factor (IU/mL)**	*r*=	0.511	−.448
	*P* value	0.008*	0.001*
**Serum cystatin C (mg/L)**	*r*=		− 0.766
	*P* value		0.001*

*P was significant if < 0.05. *r* = correlation

## Discussion

Renal function testing is important in monitoring and predicting the mortality in patients with chronic hepatitis with cirrhosis as in hepatorenal syndrome [23]. AKI is the most common extrahepatic manifestation associated with increasing mortality in patients with acute-on-chronic liver failure and often occurs with the serum creatinine level within the normal range, and the authors concluded that minor increases in serum creatinine are clinically relevant and can adversely affect survival [23, 24]. HCV may induce chronic liver fibrosis and kidney injury or hepatorenal syndrome through either direct viral invasion of the renal parenchyma [25, 26], glomerular immune complex deposition, renal complications of its extrarenal hepatic manifestations [27], or nephrotoxicity of drugs used for its treatment [3, 4]. Serum cystatin C level was an indirect marker of liver fibrosis in several chronic liver diseases [8, 28].

Therefore, our aim was to assess the biomarkers helpful for the diagnosis of early renal dysfunction by testing for cryoglobulins, RF, complement C3, cystatin C, and creatinine/cystatin C ratio in newly diagnosed HCV-infected patients.

Cryoglobulins are immunoglobulins that precipitate or form a gel when exposed to temperatures below 37°C and re-solubilize when re-warmed in vitro [17]. Mixed cryoglobulins are potentially present during connective tissue and autoimmune diseases and chronic infections [25]. Mixed cryoglobulins can cause renal injury in almost 30% of cases with nephropathy called cryoglobulinemic glomerulonephritis [27]. Mixed cryoglobulins lead to chronic renal failure in 14% of cases within 6 years [29]. The kidney is one of the most easily involved organs in HCV-infected carries with risk for AKI such as cryoglobulinemic vasculitis and glomerulonephritis [30, 31].

Our study illustrated a significant difference in cryoglobulin concentration between groups IIa and IIb, while in the control group cryoglobulins were undetected and in groups IIa and IIb the level of cryoglobulins was significantly increased. This finding may be supported by many studies that reported the relationship between HCV infection and cryoglobulin formation [32–35].

The complement system is part of the innate and acquired immunity programs that clear pathogen components from an organism [36]. Therefore, complement C3, an acute-phase protein, is decreased in chronic HCV patients [14, 15, 37]. Complement C3 protein plays a pivotal role in both classical and alternative pathways of complement activation [37]. The complement system is involved in the pathogenesis of a variety of liver disorders, including viral hepatitis, liver injury and repair, fibrosis, alcoholic liver disease, and liver ischemic/reperfusion injury [37].

Our data indicated a statistically significant decrease in complement C3 concentration in groups IIa and IIb compared to the control group. This finding may be supported by many investigators who reported the HCV infection reduced serum complement C3 [38–41]. Serum complement C3 levels were depleted in HCV-infected cirrhotic patients [15]. Complement activation leads to a plethora of cellular responses ranging from apoptosis to opsonization [14, 36].

Polyarthritis as an extrahepatic manifestation involving small joints, which resembles rheumatoid arthritis, has been described in association with HCV infection [16]. Also, mixed cryoglobulins comprise an IgM monoclonal component that displays a rheumatoid factor activity capable of reacting with intact IgG and/or its F(ab)2′ fragment [42–44]. RF may be present in up to 50–85% of chronic HCV patients [16, 20].

Our data indicated a statistically significant increase in RF concentration in groups IIa and IIb compared to the control group. This finding is supported by many researchers who reported the relationship between HCV infection and higher RF [20, 45].

Cystatin C is a non-glycosylated protein and freely filtered at the glomerulus, but it is metabolized in the proximal tubules so its clearance cannot be calculated [46]. Moreover, cystatin C has a shorter half-life [47] and provided an early prediction of kidney dysfunction in coronary heart diseases in spite of a normal serum creatinine level [48]. Serum cystatin C is increased with the progression of chronic viral hepatitis C as a potential marker for liver inflammation and fibrosis [8, 9, 28].

Our data indicated a statistically significant increase in cystatin C concentration in both groups IIa and IIb as compared to the control group. HCV-infected patients had significantly higher serum cystatin C (> 1.24 mg/L) and lower creatinine/cystatin C ratio (< 70.1 μMol/mg) and was correlated with renal function and extrahepatic cryoglobulins and RF and complement C3 parameters. This finding may be supported by many studies that reported the relationship between HCV infection and serum cystatin C [8–10]. The creatinine/cystatin C ratio or creatinine/cystatin C × 100 was an indicator to sarcopenia index (SI) for the prediction of liver injury and sarcopenia in liver disease [22] and monitoring the progression of non-alcoholic fatty liver disease [21]. Therefore, serum cystatin C may be more sensitive than serum creatinine in detecting earlier stages of renal dysfunction among HCV-infected individuals. Also, the elevated serum cystatin C found in the present study may be either related to hepatic and/or renal affection in HCV-infected patients with normal renal function test.

## Conclusion

HCV is an infectious disease that causes acute hepatitis C and may progress to chronic HCV manifested by increased serum total bilirubin, ALT, and AST and reduced serum albumin. Our HCV patients had many causes and not only a sign of renal impairment with slightly increased serum creatinine and BUN levels, but their levels were still within normal range. HCV-infected patients had an increased level of urine albumin/creatinine ratio and reduced creatinine clearance levels. Our HCV patients have many causes and not only a sign of renal impairment, and their analysis was not out of the normal range. The extrahepatic manifestations of HCV lead to increase serum cystatin C and cryoglobulins and RF levels but also reduced creatinine/cystatin ratio and complement C3 levels. Higher cystatin C (> 1.24 mg/L) or lower creatinine/cystatin C ratio (< 70.1 μMol/mg) was correlated with renal function and extrahepatic laboratory investigations in newly HCV-infected patients with normal renal function test.

### Limitations of study

More advanced and expanded studies are recommended on a larger number of patients including their clinical data and complaints to get a better evaluation and to clarify the correlation of cystatin C in patients with HCV on early renal impairment (clinical and advanced renal investigatory parameters) as extrahepatic manifestation.

## Data Availability

The datasets used and/or analyzed during the current study are available from the corresponding author on reasonable request.
